# How COVID-19 Patients Were Moved to Speak: A Rehabilitation Interdisciplinary Case Series

**DOI:** 10.1007/s11420-020-09778-0

**Published:** 2020-08-26

**Authors:** Brianne Mooney, Cecelia Lawrence, Elizabeth Gerosa Johnson, Amanda Slaboden, Karen Ball

**Affiliations:** Department of Rehabilitation, Hospital for Special Surgery, 535 70th E. St, New York, NY 10021 USA

**Keywords:** coronavirus, COVID-19, speech language pathology, physical therapy, ICU rehabilitation, tracheostomy

## Abstract

**Background:**

Up to 36% of patients admitted to the ICU for COVID-19 require tracheostomy. While the literature recommends the use of multidisciplinary teams in the management of patients with tracheostomy for other diseases, little is known on the collaborative administration of physical therapy and speech language pathology services in the COVID-19 population.

**Purpose:**

We sought to determine the outcomes of a collaboration between physical therapy (PT) and speech language pathology (SLP) in the treatment of patients who underwent tracheostomy placement as part of their treatment for COVID-19 at our facility.

**Methods:**

We conducted a retrospective case series on patients with COVID-19 who had a tracheostomy. We included patients who had undergone mechanical ventilation for 14 days or longer, had a surgical tracheostomy, been discharged from intensive care to a medical unit, and received PT and SLP referrals. We compiled retrospective data from electronic medical records, analyzing days from tracheostomy to achievement of PT and SLP functional milestones, including mobility, communication, and swallowing. Of six critically ill patients with COVID-19 who had tracheostomy placement at our facility, three met inclusion criteria: patient 1, a 33-year-old woman; patient 2, an 84-year-old man; and patient 3, an 81-year-old man. For all patients, PT interventions focused on breathing mechanics, secretion clearance, posture, sitting balance, and upper and lower extremity strengthening. SLP interventions focused on cognitive reorganization, verbal and nonverbal communication, secretion management, and swallowing function. Intensity and duration of the sessions were adapted according to patient response and level of fatigue.

**Results:**

We found that time to tracheostomy from intubation for the three patients was 23 days, 20 days, and 24 days, respectively. Time from tracheostomy insertion to weaning from ventilator was 9 days for patient 1, and 5 days for patient 2 and patient 3. Regarding time to achieve functional PT and SLP milestones, all patients achieved upright sitting with PT prior to achieving initial SLP milestone of voicing with finger occlusion. Variations in progression to swallowing trials were patient specific and due to respiratory instability, cognitive deficits, and limitations in production of an effortful swallow. Patient participation in therapy sessions improved following establishment of oral verbal communication.

**Conclusion:**

Interdisciplinary cooperation and synchronized implementation of PT and SLP interventions in three COVID-19 patients following prolonged intubation facilitated participation in treatment and achievement of functional milestones. Further study is warranted.

**Electronic supplementary material:**

The online version of this article (10.1007/s11420-020-09778-0) contains supplementary material, which is available to authorized users.

## Introduction

COVID-19, the disease caused by the novel coronavirus (SARS-CoV-2), originated in Wuhan, China, in December 2019. As of May 14, 2020, there were 4,307,287 cases worldwide and nearly 300,000 deaths [6]. The majority of people with COVID-19 present with mild symptoms and do not require hospitalization. Approximately 26% do require hospitalization, and of those 14 to 20% require intensive care unit (ICU) admission [15]. Among ICU admissions for COVID-19, nearly one third present with acute respiratory distress syndrome (ARDS), 13% with acute cardiac injury, and 7.9% with acute kidney injury [16]. Prolonged intubation and high rates of reintubation are reported in COVID-19 ICU cases in the USA [8, 20]. During the height of the COVID-19 pandemic in New York City, there was increasing demand for ventilators and ICU beds for patients with COVID-19. In order to help meet this demand, Hospital for Special Surgery (HSS) converted operating rooms and a recovery unit into ICUs. From April 2 to May 20, 2020, 26 critical care patients who required mechanical ventilation were admitted to the ICU at HSS from NewYork-Presbyterian Hospital’s ICU.

Bhatraju et al. found that during the first 7 days of mechanical ventilation for COVID-19, 77% of patients had pulmonary secretions categorized as moderate or thick and purulent [4]. While high secretion load is not common in the majority of cases, it appears to be a prominent feature in those requiring intubation and a principal factor in failure to wean. Often a tracheostomy is performed to enable weaning from prolonged mechanical ventilation [5]. The tracheostomy assists with secretion clearance through the use of suctioning and allows for a reduction in sedation use, thus facilitating weaning from mechanical ventilation [1]. Angel et al. reported that 36% of COVID-19 patients admitted to the ICU underwent a tracheostomy, with an average time to tracheostomy of 10.6 days [2]. Similar percentages are reported in patients with ARDS, 13% of whom undergo a tracheostomy, with a median time to tracheostomy of 14 days [2].

Literature suggests that there is significant benefit of multidisciplinary team (MDT) management of patients with tracheostomies and decannulation [4, 17]. For example, in oral secretion management, physical therapists focus on positioning, mobilization, ventilation, and sputum management; speech language pathologists focus on the ability to manage oral secretions and provide therapeutic exercises aimed at increasing patient awareness and clearance of saliva [9]. While roles and benefits are clearly defined for patients with tracheostomies, there are few studies describing benefits of collaboration between physical therapy (PT) and speech language pathology (SLP) in overall treatment of patients with ARDS and none to our knowledge on a collaborative approach to treatment of patients with COVID-19. Given reported benefits of MDT in optimizing outcomes in patients with tracheostomies, PT and SLP teams selected a collaborative approach to treat patients with confirmed COVID-19 and tracheostomy at HSS to achieve functional milestones.

This retrospective case series examines patients with COVID-19 who received mechanical ventilation for 14 days or longer, required tracheostomy placement, and received collaborative PT and SLP services. We sought to determine how the collaborative therapy approach was implemented and whether it had an effect on time to achieve functional milestones for both PT and SLP interventions.

## Case Summaries

This retrospective case series was approved by the Institutional Review Board at HSS and was conducted from April 4, 2020, to May 30, 2020. We retrospectively collected data from electronic medical records of critically ill patients with COVID-19 who had undergone tracheostomy at HSS. There were six patients in all and three who met the inclusion criteria: mechanical ventilation of 14 days or longer, surgical tracheostomy, discharge from the ICU to a telemetry medical floor, and PT and SLP referrals. Exclusion criteria were ICU transfer to another institution and death. The three patients who received a tracheostomy and were excluded from the study had been transferred to another institution for ongoing critical care. Patient characteristics are listed in Table 1.

**Table 1:** Patient characteristics

	Case 1	Case 2	Case 3
Age (years)	33	81	84
Gender	Female	Male	Male
Preferred language	English	English	English
Body mass index (kilograms/meter²)	29.38	25.56	29.51
Race	White	White	White
Ethnicity	Not Hispanic or Latino	Not Hispanic or Latino	Not Hispanic or Latino
Comorbidities	Left tib-fib fracture	Metastatic prostate Ca, CLL, B/L sensorineural hearing loss, hypothyroidism, asthma	Prostate Ca with Lung Mets, TURP
Symptoms on admission	Fever, SOB, cough, hypoxia SpO2 88%, tachypnea	Cough, fever, SOB, hypoxia SpO2 90% on 6L O2	2 day history of SOB, cough, diarrhea
Admission date to outside hospital	3/21/20	3/27/20	3/23/20
Admission date to HSS	4/4/20	4/2/20	4/3/20
Date of tracheostomy insertion	4/13/20	4/16/20	4/16/20
Post ICU medical issues	Ileus, bleeding from trach stoma, nausea and emesis, RUE myopathy/neuropathy, anxiety, tachycardia, ICU delirium	High sputum load, stridor, constipation, hyponatremia, sacral decubitus, ICU delirium	Toxic metabolic encephalopathy and subacute stroke, lower GI bleed, segmental PEs, hypercalcemia, tachycardia, slow to wean O2

*Tib-Fib* tibia and fibula, *SOB* shortness of breath, *ICU* intensive care unit, *RUE* right upper extremity, *Ca* cancer, *CLL* chronic lymphocytic leukemia, *SpO2* peripheral capillary oxygen saturation, *L* liters, *B/L* bilateral *O2* oxygen, *Lung Mets* lung metastases, *TURP* transurethral resection of prostate, *GI* gastrointestinal, *PE* pulmonary embolus, *Trach* tracheostomy

### Patient 1

A 33-year-old woman without significant past medical history (PMH) was admitted to another hospital on March 21, 2020, with hypoxia and shortness of breath and intubated the same day. On April 4, she was transferred to the ICU at our facility, where she exhibited an altered mental status (AMS) with agitation upon sedation weaning. Multiple unsuccessful attempts to wean from the ventilator resulted in tracheostomy and nasogastric tube (NGT) placement on April 13. Due to increased opacification of lung fields, difficulty with secretion clearance, and persistent agitation, she required 9 additional days of mechanical ventilation. Following ventilator weaning, she exhibited signs of delirium, as well as ongoing nausea and emesis that dissipated upon NGT removal. Additional signs and symptoms included right upper extremity myopathy/neuropathy, anxiety, and bleeding from the tracheostomy stoma site.

### Patient 2

An 81-year-old man with PMH of metastatic prostate cancer, asthma, and chronic lymphocytic leukemia was admitted to another hospital on March 27, 2020, with hypoxia and shortness of breath; he was subsequently intubated. He was transferred to our ICU on April 2, exhibiting AMS, generalized weakness, and poor secretion management. Multiple failed attempts to wean from the ventilator resulted in tracheostomy and NGT placement on April 16. The NGT was removed on April 23 and replaced with a percutaneous endoscopic gastrostomy (PEG) tube. He required 5 additional days of mechanical ventilation before being successfully weaned from the ventilator. Post-weaning, he produced thick, purulent secretions requiring frequent suctioning; he also developed a pressure ulcer, constipation, delirium, and respiratory stridor.

### Patient 3

An 84-year-old man with PMH of prostate cancer with lung metastasis was admitted to another hospital on March 23, 2020, with shortness of breath and cough and subsequently intubated. On April 3, he was transferred to our ICU. He was not weaned from the ventilator due to an inability to maintain ventilator synchrony, in addition to AMS. Consequently, a tracheostomy and NGT placement occurred on April 16, and the NGT was replaced with a PEG tube on April 23. He was successfully weaned from the ventilator 5 days post-tracheostomy. Thereafter, his mental status and physical function improved, but on April 26 his level of arousal and physical capacity deteriorated and he was diagnosed with toxic metabolic encephalopathy. Magnetic resonance imaging performed on May 5 also demonstrated signs of a subacute stroke. Following discharge from the ICU to the medical floor, he was found to have pulmonary embolism, which delayed his weaning from supplemental oxygen.

### Interventions

PT and SLP developed treatment guidelines based upon international consensus guidelines, available research regarding COVID-19 disease presentation, and clinical knowledge of therapeutic interventions for disease processes similar to ARDS [9, 14, 19]. Patients were screened daily from ICU admission during interdisciplinary morning rounds for factors such as medical stability, ventilator requirements, medical and nursing goals for the day, and potential procedures. A treatment plan was established. Cognitive status including patients’ ability to follow commands dictated what type of interventions was performed. Additionally, initiation of a communication and swallowing intervention required ability to follow simple commands, tolerate upright positioning, elicit an effective cough, and produce voice.

**Fig. 1:**
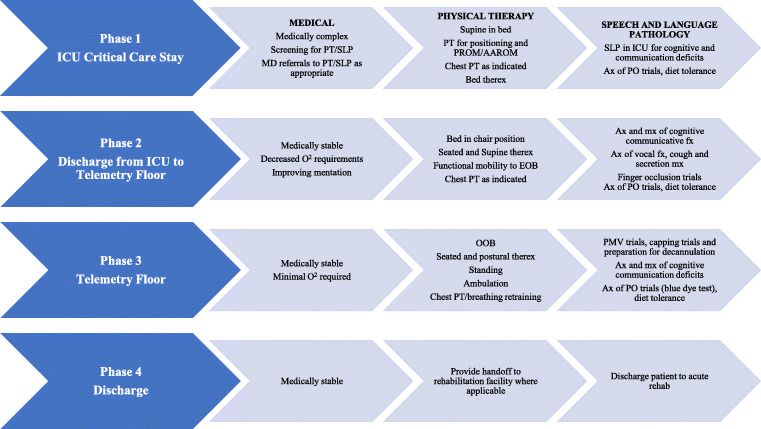
PT and SLP phases of intervention. *PT* physical therapy, *SLP* speech language pathology, *ICU* intensive care unit, *PROM* passive range of motion, *AAROM* active-assisted range of motion, *PO* per oral *O2* oxygen, *EOB* edge of bed, *Ax* assessment, *Mx* management, *Fn* function, *OOB* out of bed, *Therex* therapeutic exercise, *PMV* Passy-Muir valve, *Acute Rehab* acute rehabilitation facility

A four-phase inpatient approach for PT and SLP was implemented for the critically ill COVID-19 patients admitted to the ICU (Fig. 1). Post-tracheostomy, patients were seen daily by PT. After being weaned from the ventilator, those who met SLP criteria for treatment received therapy four or five times weekly. Average length of PT and SLP sessions was 60 min and 45 min, respectively. Patients transitioned through the phases of treatment depending on response to treatment as determined by vital signs (stable blood pressure, heart rate, respiratory rate, and oxygen saturation) (Fig. 1). The Modified Borg Scale (MBS) is commonly used in cardiopulmonary populations as a measure of perceived exertion and is recommended for use in the COVID-19 patients [10, 11]. While we used the MBS and patient subjective fatigue levels as a means of assessment and progression, due to the need for rapid adaptation of services, these values were not consistently documented in the electronic record for these three cases. Progression in SLP treatment was determined via clinical bedside assessment and informed clinical opinion of the treating clinician.

PT interventions focused on breathing mechanics, secretion clearance, posture, sitting balance, and upper and lower extremity strengthening in order to optimize functional mobility. SLP interventions focused on cognitive reorganization, establishment of verbal and nonverbal communication, secretion management, and swallowing function. Initial voicing attempts were facilitated by finger occlusion—covering the tracheostomy to redirect airflow through the upper respiratory system. SLP intervention also targeted swallowing, as there is a correlation between prolonged intubation and moderate to severe dysphagia, with recommendations for early SLP involvement in these cases [12]. Among patients with a tracheostomy, there are many factors that can further complicate swallowing, such as differences in airflow, hyolaryngeal excursion, vocal fold mobility, and cuff inflation [3, 7]. Based on patient feedback and analysis of progression, we found it would be beneficial for the two disciplines to coordinate care in order to optimize outcomes, especially because participation in therapy was affected by the patient’s level of fatigue. PT and SLP sequentially scheduled PT prior to SLP to optimally position patients, address breathing mechanics, and promote secretion clearance. Intensity and duration of the sessions were continuously adapted within and between sessions, according to patient response and level of fatigue, to optimize performance and facilitate return to function.

### Time to Tracheostomy Milestones

Time to tracheostomy from intubation for the three cases was 23 days, 20 days, and 24 days, respectively, for patients 1, 2, and 3. Time from tracheostomy insertion to complete weaning from the ventilator was 9 days for patient 1 and 5 days for patients 2 and 3. Downsizing of tracheostomy to a cuffless size 6 tube occurred 22 days after insertion for patients 1 and 2 and 32 days for patient 3. All three patients received downsized tracheostomy tubes within a week of achieving voicing through finger occlusion or Passy-Muir Valve (PMV) use. Furthermore, Patient 1 was able to be decannulated 28 days after initial insertion of tracheostomy. Average length of mechanical ventilation for the three cases was 29 days, proving to be a prolonged period of intubation for the COVID-19 population.

### Time to Achieve Functional PT and SLP Milestones

Table 2 depicts time to achieve functional milestones for PT and SLP for each patient. Of note, each of the three patients sat upright with the bed in chair position with PT prior to achieving the initial SLP milestone of voicing with finger occlusion. Achievement of variations in progression to swallowing trials was patient specific and due to respiratory instability, cognitive deficits, and hypophonic voice, as well as limitations in production of an effortful swallow and volitional cough*.*

**Table 2:** Days to achievement of PT and SLP milestones from date of tracheostomy insertion

	Case 1			Case 2			Case 3		
	Medical	PT	SLP	Medical	PT	SLP	Medical	PT	SLP
Mechanical ventilation (total)	32			25			30		
Mechanical ventilation (trach)	9			5			5		
Trach downsize	22			22			32		
Decannulation	28			N/A			N/A		
LOS (ICU)	34			27			32		
LOS Total	55			49			58		
Bed mobility max assist		2			4			27	
Bed mobility min assist		10			16			N/A	
Bed mobility independent		14			26			N/A	
Upright sitting bed in chair position		11			9			29	
Sitting EOB supported		16			10			N/A	
Sitting EOB unsupported		17			20			N/A	
Sitting OOB		22			13			33	
Standing		20			26			N/A	
Ambulation		27			N/A			N/A	
Voicing with finger occlusion			18			15			31
Voicing with PMV			24			22			35
Capping			26			23*			N/A
Volitional cough			19			17			31
Ice chips			19			17			N/A
Blue dye			24			N/A			N/A
PO diet			25			N/A			N/A

*PT* physical therapy, *SLP* speech language pathology, *Trach* tracheostomy, *LOS* length of stay, *ICU* intensive care unit, *Max* maximum, *Min* minimum, *EOB* edge of bed, *OOB* out of bed, *PMV* Passy-Muir valve, *PO* per oral, *N/A* not applicable

^*^Patient failed capping trial

## Discussion

We describe three patients with COVID-19 who received coordinated PT and SLP following prolonged intubation and tracheostomy. All three patients achieved various functional milestones; the case findings suggest a positive impact of collaborative treatment. With assistance of PT to properly position patients, facilitating improved strength and endurance and maximizing respiratory functioning, SLP was able to significantly improve swallowing and verbal communication, which in turn improved participation in PT.

Limitations of this study include its retrospective, observational design, and its small sample size; statistical analysis was therefore not possible. These limitations also make it difficult to generalize results about the effectiveness of coordinated PT and SLP. As a result of interinstitutional patient transfer, we had limited data on the previous medical and therapeutic interventions for these patients. Knowledge of the three patients’ treatment prior to transfer to our facility would have allowed us to assess its effects on patient outcomes.

An important example of the potentially positive impact of collaborative care is that given the uniformity of the size and type of the patients’ tracheostomies, voicing through air leak and finger occlusion was more challenging and unlikely; the outer cannula takes up space within the airway as does the deflated cuff, limiting airflow to the vocal folds. SLP required PT assistance to position the patient upright so that respiratory support could be achieved. This facilitated vocal fold movement and enabled SLP to accurately assess voicing ability. Without the proper positioning of the patients, it would have been significantly more difficult for patients to achieve voice, thereby impacting SLP ability to accurately assess the patient’s ability to voice or ready the patient for voice trials. This was crucial to determine readiness for verbal communication as well as airway protection for swallowing function. Therefore, it was mutually beneficial for the two disciplines to coordinate care in order for the patients to yield best outcomes for both respective therapy sessions.

**Fig. 2:**
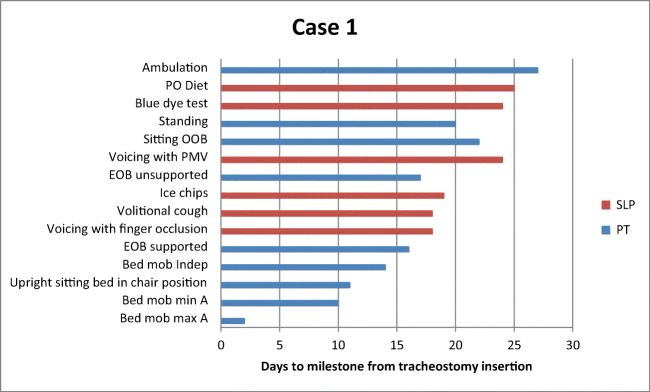
Case 1 days to achievement of functional milestones from tracheostomy insertion. *PO* per oral, *OOB* OUT OF BED, *PMV* Passy-Muir valve, *EOB* edge of bed, *Mob* mobility, *Indep* independent, *Min A* minimal assist, *Max A* maximal assist, *PT* physical therapy, *SLP* speech language pathology

**Fig. 3:**
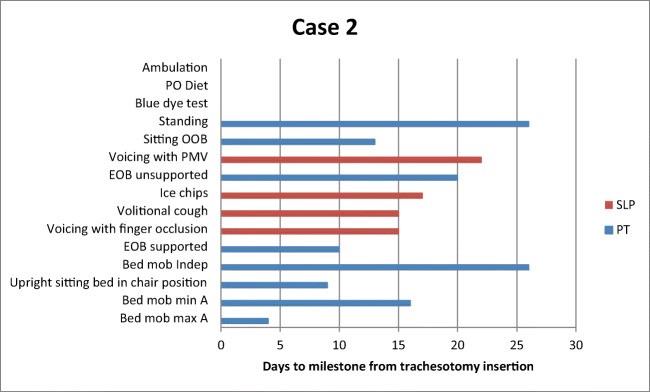
Case 2 days to achievement of functional milestones from tracheostomy insertion. *PO* per oral, *OOB* out of bed, *PMV* Passy-Muir valve, *EOB* edge of bed, *Mob* mobility, *Indep* independent, *Min A* minimal assist, *Max A* maximal assist, *PT* physical therapy, *SLP* speech language pathology

**Fig. 4:**
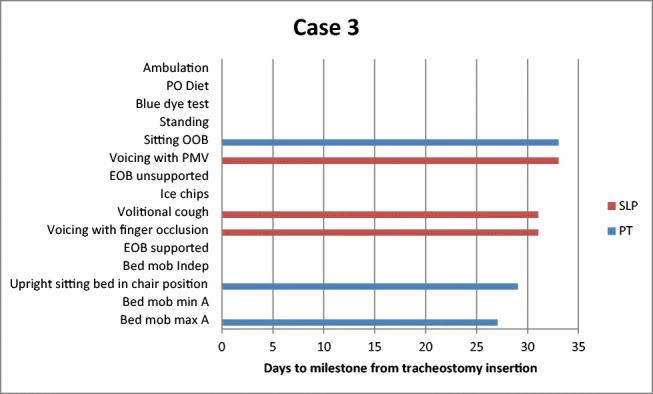
Case 3 days to achievement of functional milestones from tracheostomy insertion. *PO* per oral, *OOB* out of bed, *PMV* Passy-Muir valve, *EOB* edge of bed, *Mob* mobility, *Indep* independent, *Min A* minimal assist, *Max A* maximal assist, *PT* physical therapy, *SLP* speech language pathology

The timing of PT intervention in preparation for SLP intervention maximized improvement of function of both physical and speech milestones (Figs. 2, 3, and 4). Anecdotally, the authors felt timing also maximized patient morale as patients appeared more motivated and were better able to participate directly in their care. Following establishment of consistent vocalizing, patients’ willingness to participate increased in both PT and SLP sessions. This in turn reduced patients’ frustration and facilitated reorientation, which helped with delirium. It should be noted that all patients preferred voicing over non-verbal options such as communication boards, despite initial failed attempts at voicing. This is consistent with previous research that found patients often prefer using their voice to communicate, notably if they had access to their voice prior to tracheostomy [21]. By improving strength and sitting posture, patients increased respiratory capacity and participation in activities. In addition, improved posture and increased upper extremity strength and mobility facilitated attempts at finger occlusion. Finger occlusion provided increased voicing trials and facilitated improved cough, which assisted with airway protection and led to greater independence with verbal communication. Increased independence and motivation to participate improved efforts to achieve functional milestones and participation in more frequent therapy sessions. Finally, progress in therapy improved patients’ quality of life through the ability to communicate with family members through FaceTime.

Early PT and SLP intervention was often hampered by fluctuations in patients’ medical and cognitive status, suboptimal positioning in bed, reduced trunk control, and generalized weakness. Following supportive intervention with PT, successful SLP sessions facilitated voicing through finger occlusion and promoted communication which led to eventual downsizing and change of tracheostomy to cuffless and maximized PMV success, which is crucial for swallowing safety [18].

The use of MDT in management of patients with a tracheostomy is well documented; however, currently there is no literature on MDT use in COVID-19 patients that addresses the impact of collaborative and ordered intervention of PT and SLP on the motor system [5]. Clinical guidelines indicate that PT intervention is beneficial and safe in critically ill patients with COVID-19 [22]. This case series supports this theory, as all three patients appeared to benefit from PT (Table 2). Guidelines on SLP interventions for patients with COVID-19 recommend limiting in-person care due to the aerosol-generating procedures in swallowing assessment and treatment [14, 24]. However, for urgent management of swallowing disorders, such as patients with tracheostomies following prolonged intubation, SLP intervention is indicated with appropriate PPE and safety considerations. [12] Anecdotally, due to the isolation required for patients with COVID-19, researchers felt patients’ demeanor and affect during sessions improved with in-person SLP intervention as patient were more engaged in therapy.

Several salient features of COVID-19 appeared to affect patients’ ability to progress with therapy. First, we noticed high levels of fatigue hospital-wide in COVID-19 patients with varying disease severity, significantly affecting their tolerance for therapy. AMS during and after weaning from the ventilator also affected progress in PT and SLP. AMS was likely due to COVID-19-induced hypoxia, prolonged intubation, and consequent need for sedation. One patient developed toxic metabolic encephalopathy and subacute stroke after ventilator weaning, significantly impairing his ability to participate in early therapy. While central nervous system COVID-19 deficits are investigated in the literature, the effect on rehabilitation is not documented as far as we know [13].

While timeliness of referral was not an initial area of concern, it can be argued that earlier PT and SLP interventions may have improved functional outcomes, as patient engagement in PT intervention peaked with onset of successful communication. Therefore, education of interdisciplinary teams regarding PT and SLP roles is vital to promote earlier intervention in critically ill patients with COVID-19. In order to maximize therapist safety when treating patients with COVID-19, institutions should have a clear, comprehensive contingency plan for the management patients with COVID-19 [19, 23].

This case series highlights the positive effects of collaborative efforts between PT and SLP in the management of three critically ill patients with COVID-19 who underwent tracheostomy. Future research on discipline-specific interventions and collaboration between PT and SLP is warranted. Further study is also needed on the education and practice of interdisciplinary teams, on the identification of patients who would benefit from collaborative interventions, on the timing of such interventions, and on the effects of collaboration between PT and SLP in patients with COVID-19 using patient-reported outcome measures.

## Electronic supplementary material

ESM 1 (PDF 1224 kb)

ESM 2 (PDF 1224 kb)

ESM 3 (PDF 1224 kb)

ESM 4 (PDF 1224 kb)

ESM 5 (PDF 1224 kb)
